# Chemokine Receptors and Exercise to Tackle the Inadequacy of T Cell Homing to the Tumor Site

**DOI:** 10.3390/cells7080108

**Published:** 2018-08-17

**Authors:** Manja Idorn, Per thor Straten

**Affiliations:** 1 Center for Cancer Immune Therapy, Herlev Gentofte University Hospital, Herlev Ringvej 75, 2730 Herlev, Denmark; 2 Department of Immunology and Microbiology, University of Copenhagen, Blegdamsvej 3B, 2200 Copenhagen, Denmark; per.thor.straten@regionh.dk

**Keywords:** adoptive cell therapy, ACT, tumor infiltrating lymphocytes, TIL, genetic engineering, CARs, exercise, chemokines, homing

## Abstract

While cancer immune therapy has revolutionized the treatment of metastatic disease across a wide range of cancer diagnoses, a major limiting factor remains with regard to relying on adequate homing of anti-tumor effector cells to the tumor site both prior to and after therapy. Adoptive cell transfer (ACT) of autologous T cells have improved the outlook of patients with metastatic melanoma. Prior to the approval of checkpoint inhibitors, this strategy was the most promising. However, while response rates of up to 50% have been reported, this strategy is still rather crude. Thus, improvements are needed and within reach. A hallmark of the developing tumor is the evasion of immune destruction. Achieved through the recruitment of immune suppressive cell subsets, upregulation of inhibitory receptors and the development of physical and chemical barriers (such as poor vascularization and hypoxia) leaves the microenvironment a hostile destination for anti-tumor T cells. In this paper, we review the emerging strategies of improving the homing of effector T cells (TILs, CARs, TCR engineered T cells, etc.) through genetic engineering with chemokine receptors matching the chemokines of the tumor microenvironment. While this strategy has proven successful in several preclinical models of cancer and the strategy has moved into the first phase I/II clinical trial in humans, most of these studies show a modest (doubling) increase in tumor infiltration of effector cells, which raises the question of whether road blocks must be tackled for efficient homing. We propose a role for physical exercise in modulating the tumor microenvironment and preparing the platform for infiltration of anti-tumor immune cells. In a time of personalized medicine and genetic engineering, this “old tool” may be a way to augment efficacy and the depth of response to immune therapy.

## 1. Introduction

Within the past decade, immune therapy has revolutionized the treatment of cancer and changed the outlook for patients with metastatic disease. The term “Immune Therapy” denotes approaches aimed at modulating the immune system to target and kill cancer cells and can be boiled down to three distinct treatment strategies: (1) Vaccines immunizing against tumor antigens (TAs), (2) Adoptive cell therapy (ACT) of *ex vivo* expanding immune effector cells, and (3) immune modulators improving endogenous anti-tumor immunity [1,2].

TA-specific T cells are readily found in the blood of patients with cancer and these cells infiltrate tumors despite having limited efficacy. Thus, tumors are infiltrated with tumor-reactive T cells but, in most cases, at low frequency [3]. To this end, a high frequency of tumor infiltrating of lymphocytes (tumor infiltrating lymphocytes, TIL) such as in CD8^+^ T cells have been associated with improved survival of patients of several cancer diagnoses including melanoma [4], ovarian [5], breast [6], and colorectal cancer [7,8] even though solid prospective phase III clinical data are still missing. In addition, accumulating data supports the notion that baseline tumor infiltration by activated CD8^+^ T cells (inflamed tumors) identifies a group of patients with a better chance for a clinical response to treatment with immunotherapy when compared to patients with non-inflamed tumors [9,10]. Thus, even though T cells can recognize and kill tumor cells, recruitment and infiltration of TA-specific T cells to tumors seems to impact overall survival and also represents a denominator for response to therapy using check point inhibitory antibodies.

Chemokines responsible for TIL recruitment and migration to the tumor site are found among the pro-inflammatory chemokines in both metastatic melanoma (MM) and ovarian cancer (OC), which associates with a TIL inflamed phenotype [1,11,12,13]. In addition, expression and binding of specific chemokine/chemokine receptors such as CXCR3-CXCL9/CXCL10 have been proposed as non-redundant requirements for endothelial transmigration of T cells across tumor vasculature in melanoma [14]. Consistent with this, melanomas with low expression of ligands for chemokine receptors CXCR3 and CCR5 are poorly infiltrated [1]. 

In addition, other roadblocks may occur on the anti-tumor T cell’s path to the tumor site among other abnormal vascularization, poor perfusion, presentation of appropriate adhesion molecules, and hypoxia. Discussing old data of exercise physiology and new data on the effect of exercise on cancer, we propose a potential role for exercise in improving cancer immune therapy.

### Adoptive Cell Therapy (ACT)

Chemokines capable of recruiting anti-tumor T cells are found in both metastatic melanoma (MM) and ovarian cancer (OC) and have been associated with a TIL inflamed phenotype [1,11,12,13]. Expression and binding of specific chemokine/chemokine receptors such as CXCR3-CXCL9/CXCL10 have been proposed as non-redundant requirements for endothelial transmigration of T cells across tumor vasculature in melanoma [14]. However, these chemokines are not expressed in all patient tumors from these indications. In fact, far from it. Consistent with this, melanomas with low expression of ligands for chemokine receptors CXCR3 and CCR5 are poorly infiltrated [1].

ACT is a strategy in which immune effector cells are expanded through *ex vivo* and transferred back to the patient. Most strategies take advantage of autologous cells and, in most cases, T cells but NK cells are studied in ACT as well. Moreover, some strategies are based on the genetic engineering of cells for the generation of tumor specific cells. Alternatively, naturally elicited tumor-specific cells may be harvested from the patient and expanded for ACT. Patients may receive preconditioning therapy (lymphodepletion) prior to ACT and cytokine support-typically IL-2—may be used to support the survival of transferred cells upon transfer [15,16,17]. 

TIL from tumor biopsies has reproducibly generated high response rates and durable, complete responses in patients with MM [16,17,18]. As reported in a paper from Rosenberg et al. 2011 [16], the overall response and complete response rates were approximately 50% and 20%, respectively. A total of 95% of complete responders were without evidence of disease at their five-year follow-up. 

Response to TIL therapy highly depends on the quantity and quality of the infused T cells. Several studies have correlated with the overall quantity of infused TILs by favoring a high CD8^+^ count [19,20]. In addition, TIL with a phenotype of early differentiation state with high CD27 expression and longer telomeres [17,21] *in vitro* and *in vivo* tumor reactivity (PDX mouse model) [17,18,22,23] correlates with and predicts response to TIL therapy. As such, one could speculate that improving the starting (biopsy) material-quantity and quality of T cells in tumors harvested for TIL expansion-could improve the infusion product and efficacy of therapy. This is discussed later in the manuscript.

As of yet, limited data are available concerning TIL therapy beyond the treatment of MM. Moreover, as a source of biological material for clinical use, TIL is highly heterogeneous. In addition, not all cancers are surgically accessible or generates TIL (haematological cancers). Thus, alternative strategies have been pursued using peripheral blood T cells genetically engineered to express tumor-specific chimeric antigen receptors (CAR) or (affinity enhanced) TA-specific TCR [24,25]. ACT of CARs targeting CD19 has been very successful in treating CD19^+^ B cell malignancies [24,26]. However, as of yet, CAR therapy has not been able to achieve similar responses to solid cancers as those observed in haematological malignances. This is, in part, due to the lack of surface expression of tumor specific antigens [27]. To this end, CD19 is a lineage antigen expressed on a suspendable lineage of cells. However, in many instances, adverse killing of healthy cells expressing a lineage or tissue specific antigen could have detrimental consequences. As such, engineering T cells with TCRs of defined specificity and affinity enhanced TCRs binding to the cognate antigen enable the targeting of commonly mutated, differentiated or cancer testis antigens, which are expressed intracellularly [25]. However, besides the obvious limitation of MHC-restriction, TCR engineered T cells have been marred by some devastating adverse events in clinical trials [28,29]. Most prominent was a trial of affinity enhanced TCR to melanoma-associated antigen 3 (MAGE-A3), which killed two patients due to cross reactivity with Titin. This is a peptide found in contracting heart muscle [29,30].

The success of CAR T cells have, however, been limited to the treatment of hematologic cancers where the tumor cells circulate in the same lymphoid compartments evading the need for “adequate” homing of transfused CAR T cells [24,26]. To this end, CAR or TCR T cell therapy has been less successful in treating solid cancers located outside of the lymphoid circulation. The multifaceted hurdles impeding CAR and TCR T cell therapies have been thoroughly reviewed elsewhere [27,31,32]. However simplified, this could suggest that homing to the tumors is difficult, which will be the focus of this review. In support of this hypothesis, tumor control was achieved in animals only after local (intra-tumoral) administration of Melan-A TCR transduced T cells while i.v. administration of T cells was less efficient [33]. In fact, by in vivo imaging of fluorescently labelled T cells, the authors showed how i.v. administered T cells primarily located in the lungs, liver, and spleen. While T cells did eventually accumulate at the tumor site, the specific T cell signal in tumors corresponded to <1% of the infused T cell signal [33]. 

Only very early clinical trials of ACT have evaluated homing of T cells to the metastatic tumor sites in patients. To this end, radioactively labelled TILs were used to study the homing characteristics in MM patients treated with autologous TILs [34,35]. While radioactively labelled T cells didaccumulate at the tumor site at 48–72 h post ACT (continually increasing for 5–9 days), they were initially located in the lungs, liver, and spleen, as observed with other ACT products. While the total number of infused T cells correlated with an amplitude of a radioactive signal in the tumors and in the response to therapy, the maximum percentage of recovered injectate in the tumor was only 0.016% 10 days after ACT [34,35]. 

Taken together, this supports the notion that adequate homing of T cells to the tumor site is a rate limiting factor in successful immune therapy of solid tumors with ACT [27,31]. Tumor instigated chemokine/chemokine receptor mismatching and disrupted endothelial vessels and adhesion molecule expression are considered key factors in limiting TIL infiltration [1]. In this paper, we review efforts to address the inadequate homing of anti-tumor T cells to the tumor in the setting of ACT. In addition, we discuss the potential of “prescription” physical exercise prior to TIL harvest following the transfer to facilitate better quality and quantity of TILs in the tumor, which is a feature possibly transferrable to other immune therapy strategies (e.g., checkpoint inhibitors) as well [36,37].

## 2. Chemokines in Cellular Homing and Immune Evasion

Chemokines are chemotactic cytokines orchestrating the migration patterns and positioning of all immune cells within the body [38]. In addition to chemokine guidance, a cascade of events including the coordinated rolling and adhesion of circulating leukocytes to the endothelial surface are vital for extravasation and tissue infiltration [1]. Immune cells continuously patrol peripheral tissue and respond to migratory signals, which allows them to home to sites of inflammation [39]. Immunity against infection and cancer relies upon the ability of the immune system to circulate and patrol the entire organism. This requires the ability to sense directory ques, adhere to, and extravasate out of the bloodstream under hemodynamic flow conditions and subsequently migrate through and infiltrate infected or cancerous tissue. In a cancer setting, successful homing to a tumor is dependent on the acquisition of “tumor directing” homing receptors upon activation [1]. TA-specific T cells are activated in the LNs by recognizing tumor antigens (TA) presented by DCs, which leads to changes in the expression of surface molecules and, in turn, allowing exit from the lymph node and homing towards inflamed sites due to upregulated homing molecules, i.e., chemokine receptors specific for chemokines secreted at inflamed tissues and tumor sites [1]. This includes chemokine receptors known as CCR5 and CXCR3 as well as adhesion molecules Lymphocyte Function-associated Antigen (LFA-1) and Very Late Antigen (VLA)-1, which is important for rolling and firm adhesion to tumor vascular endothelium. Chemokines secreted by the tumors upregulate the expression of corresponding leukocyte binding molecules Intercellular Adhesion Molecule (ICAM)-1 and Vascular Cell Adhesion Molecule (VCAM)-1 [1]. Trans-endothelial migration is further aided by binding to chemokines and the recognition of TA presented by tissue MHC-Class I [1]. Once in the tumor, TA-specific T cells recognize and kill cancer cells.

Chemokines, as noted earlier, are chemotactic cytokines, which emphasizes that these molecules also have functions ascribed to cytokines including survival, proliferation, and differentiation signalling [38,40]. In the tumor microenvironment, autocrine CXCL8/IL8 signalling has been ascribed as a mechanism of survival and as a mechanism of proliferation and increased metastatic potential [41] of cancer cells expressing the cognate receptor. In addition, CXCL8/IL8 signalling has been shown to convey chemotherapy resistance in at least prostate cancer, breast cancer, and colorectal cancer [41,42,43,44]. In addition, paracrine CXCL8/IL8 signalling facilitate changes in the composition of the tumor microenvironment. CXCL8/IL8 signalling in endothelial cells is a potent pro-angiogenic factor, which, in concert with a vascular endothelial growth factor (VEGF), is pivotal for securing adequate blood supply to the growing tumor mass [13,41]. 

Additional acquisition of chemokine receptors represents a hijack mechanism by which cancer cells are capable of metastasizing to compartments of homeostatic expression of the cognate chemokines. To this end, CCR7 is associated with LN and brain metastasis in human breast cancer while CXCR4 has been ascribed to metastatic spread in brain, lungs, liver, and bones in melanoma [45], breast [46], prostate [47], and colorectal cancer [48]. Other chemokines may foster tumor progression in a paracrine manner. Focusing on CXCL12, CCL2, and CCL22, all three chemokines aid tumor development and progression by recruiting immune suppressive cell subsets. CCL2 is one of the major triggers of tumor-associated macrophage (TAMs) infiltration within the tumor microenvironment [13]. In addition, CCL2 has been shown to recruit MDSC, which are able to inhibit CD8^+^ T cell entry into the tumor microenvironment and subsequent anti-tumor response in mouse models of melanoma [49]. However, CCL2 has also been associated with a T cell infiltrated phenotype in human metastatic melanoma (MM), which complicates the understanding of CCL2 in cancer [11]. Similar to CCL2, CXCL12 recruits immune suppressive myeloid cells to the tumor site-especially MDSC [50]. CCL22 is secreted by both cancer cells and TAMs in ovarian cancers (OC) [13,51] and are responsible for the recruitment of Treg [52]. 

Thus, irrespective of whether acquisition of chemokine modulating oncogenes in cancer is an escape mechanism gained through immune selection or an early event in tumor development, tumors are able to recruit immune suppressive cells while diverting the recruitment of anti-tumor T cells. We have compared the function of chemokines in adaptive immunity and the tumor microenvironment (see Table 1). 

Limited studies have compared *in vivo* tumor homing of TILs to that of CAR or TCR after ACT. One might reason that one of the advantages of TIL therapy for solid cancer (melanoma) is explained by the use of endogenously activated T cells primed by DCs presenting tumor-derived peptides in the tumor draining lymph node. TILs have once expressed necessary tumor/tissue-specific homing receptors [1]. Thus, these cells have migrated to the tumor site, which is contrary to CAR or TCR transduced peripheral blood T cells activated *in vitro* [24,31]. However, tumor-infiltrating lymphocytes also comprise T cells of irrelevant specificity, e.g., virus specific T cells [53]. Thus, homing to sites of infections or tumors is not an antigen-specific event but rather a migration towards a site that express appropriate chemokines. We and others have seen that activation of peripheral blood T cell leads to differential expression of chemokine receptors among others CXCR3 [3,12], which are of special significance for epithelial transmigration into tumors [14,54,55].

### Exploiting Tumor-Specific Chemokine Axes

Immune escape by recruiting immune suppressive cell subsets are among the emerging hallmarks of cancer described by Hanahan and Weinberg (2011) [56] and have long been thought of as part of the complex process behind immune escape from immune surveillance [57,58]. An emerging approach of optimizing ACT has aimed at improving the homing of ACT cells by exploiting the tumor promoting chemokine/chemokine receptor axes in the tumor. This has been pursued by genetically equipping TIL, CAR, and TCR T cells with chemokine receptors matching the chemokine environment of cancer. However, even though chemokine secretion and the role of chemokines in tumor progression have been heavily reviewed [1,13,48,59,60,61,62,63,64,65,66,67], there is little original literature investigating which cells of the tumor microenvironment release individual chemokines and even less studies trying to decipher this in human cancers. Adding to the complexity of the system, the 50-odd chemokines and 20 corresponding receptors have not been systematically investigated at any one time.

Data by Harlin and colleagues [11] indicates that a wider range of chemokines is present in tumors *in vivo*. Melanoma biopsies are segregated into two major subsets such as those infiltrated by T cells and those lacking T cell infiltration-tumors with a T cell-infiltrated phenotype differentially expressed chemokines CCL2, CCL3, CCL4, CCL5, CCL19, CCL21, CXCL9, CXCL10, CXCL11, and CXCL13 whereas all tumors irrespective of the T cell infiltration level expressed detectable levels of CXCL8/IL-8 and CXCL12/SDF-1 [11]. By screening a panel of MM cell lines *in vitro*, Harlin and colleagues found that the majority secreted only a restricted set of chemokines *in vitro* including predominantly CXCL8/IL-8 and CXCL1/Gro-α [11], which are in line with studies by us and others [3,11,68,69,70,71]

Identification of chemokine receptor expression on TILs has come into focus as the issue of inadequate homing of T cells to tumors has become more obvious. As the majority of TIL trials have been performed in MM, the information below discusses findings in MM. *In vitro* expanded TILs from patients with MM have been described to express a set of chemokine receptors representing a pro-inflammatory profile including the expression of CCR5 and CXCR3 [3,72]. However, if the tumors to be targeted do not express the corresponding chemokines, the T cells will not home properly. As such, CXCR3-CXCL9/CXCL10 binging have been shown to be crucial for vascular transmigration [14] and CCR5/CCL5 and CXCR3/CXCL9/10 expression been correlated with increased T cell infiltration in MM [14]. This correlates with a response to ACT in MM [54]. Taken together, numerous chemokines are expressed in both MM and OC and, while some tumors express an extended repertoire of chemokines [11], common are the substantial secretion of CXCR2 ligands CXCL1/Gro-α and CXCL8/IL-8 and to some extent CXCL12/SDF1 and CCL2/MCP1. Of these common chemokines, the corresponding receptors were expressed on subsets of tumor-associated T cells but far from the majority of TILs from MM and OC patients [3,72]. In particular, the complete lack of CXCR2 expression on TILs makes CXCL8/IL-8 axes an ideal candidate for chemokine receptor engineering of T cells.

Kershaw et al. [68] were among the first to pursue chemokine receptor engineering of human T cells in an attempt to increase homing towards tumor-secreted chemokines [68] in MM using lentiviral transduction for permanent expression. They found CXCR2 to be a candidate and showed the feasibility of improving homing *in vitro* of TILs transduced with CXCR2. Sapoznik and colleagues [69] instead pursued mRNA electroporation of CXCR1 and CXCR2, but surface expression of CXCR2 after electroporation was disappointing and, while CXCR1 was slightly overexpressed, the expression was short-lived and peaked 18 h after transfection and reached endogenous levels after 72 h. CXCR1 overexpression, however, did increase the *in vitro* migration towards rh-IL-8 [69]. Inspired by Sapoznik and colleagues, we investigated the feasibility of mRNA electroporation of CXCR2. However, unlike their data [69], it did not experience any difficulty in reaching very high expression of CXCR2 on the surface of TILs and increasing *in vitro* migration towards rh-IL-8 [73]. mRNA electroporation has its drawbacks though. In early pilot studies of homing *in vivo*, we observed that T cell accumulation at the tumor site was dependent on the total number of infused T cells and time post tail vein administration (unpublished data). Others have observed similar trends [33,34,35,74,75] and, despite ease of use, the transient expression of the mRNA transcript proves too brief for clinical application at least for forced expression of chemokine receptors. In a xenograft mouse model of human melanoma, we recently showed that ACT of CXCR2 engineered T cells double the frequency of human CD3^+^ T cells in s.c. human melanomas, which is substantiated by the identification of CD3^+^ T cells infiltrating deep into the tumors by IHC [3]. This corroborates data by the group of Patrick Hwu who demonstrated that CXCR2-transduced T cells not only improve homing but also tumor control in an *in vivo* syngeneic mouse model [75].

While the strategy of exploiting the CXCR2 axis has been pursued by several in MM, chemokine receptor engineering has been pursued for other indications as well. We recently proved the feasibility of isolation and genetic engineering of tumor ascites lymphocytes (TALs) [72]. We found a similar disconnect between chemokines in ascites and the receptors on TALs, as we did with TILs from MM. As such, forced expression of CXCR2 on TALs improved homing towards human ovarian cancer ascites. Other chemokine/chemokine receptor pairings have been sought. To this end, CCR2 transduced CAR T cells directed towards CCL2 producing mesothelioma [74] and neuroblastoma [76] improved homing *in vitro* and mouse models. CCR4 have been used to redirect CD30 CARs towards non-Hodgkin’s lymphoma [77] and are currently being pursued in redirecting cytotoxic T cells towards CCL22 producing pancreatic cancer [78]. CXCL8/IL8 expression has been detected across several cancer indications [79] including both solid [80,81,82,83,84,85,86] and haematological malignancies [79,87,88] and thus, one might speculate, whether CXCR2 transduction might not be as efficient in these malignancies as the CCR2 and CCR4 proposed above. 

Recent data have linked the expression or mutation in several oncogenes to tumor intrinsic immune escape mechanisms. Mutation in the *RAS* oncogene-leading to constitutive signalling has been shown to induce the expression of CXCL8/IL8 in tumors [48] while others have shown that tumor intrinsic mutations in the β-catenin pathway modulates T-cell infiltration by silencing the expression of DC-recruiting chemokine CCL4 [89]. Similarly, the loss of PTEN in cancer cells significantly decreased the infiltration of T cells in mouse models of melanoma. The study showed that loss of PTEN led to an increase in CCL2 and VEGF expression and a decrease in CXCL10 [90]. In another study, VEGF was shown to be a negative regulator of transcription factor NFκB and the resultant decrease in tumor CXCL10 and 11, as a mechanism obstructing tumor T cell infiltration [91]. These data suggest that cancer cells can hijack the chemokine/chemokine receptor system through oncogenic tumor intrinsic pathways. Thus, further characterization of oncogene expression linked to specific chemokine profiles might represent a “biomarker” for the selection of personalized chemokine receptor engineering of TILs by mixing and matching the expression of one or more chemokine receptors specific for the patient tumor. 

**Figure 1:**
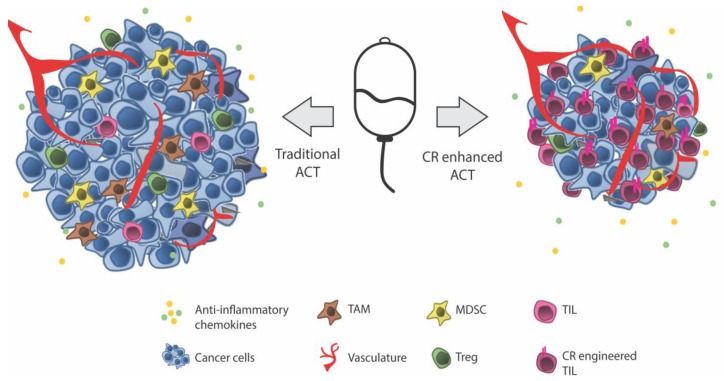
ACT of autologous *in vitro* expanded T cells (TIL, CAR and TCR T cells). ACT relies on the efficient homing of transferred T cells to the tumor site. The cells of the tumor secrete chemokines supports tumor growth and immune evasion by diverting anti-tumor immune cells. Thus, the tumor is characterized by an environment of infiltrating Tregs, TAM, and MDSC expressing inhibitory receptors and secreting immune suppressive cytokines and factors and only few dysfunctional anti-tumor T cells. (**Left**) Traditional ACT relies on endogenous chemokine receptors expressed on infused T cells to enable homing to the tumor site. (**Right**) Chemokine receptor engineered T cells, which express a selection of chemokine receptors matching the tumor chemokines, are thought to improve the homing of transferred T cells to the tumor site. As a consequence, more anti-tumor T cells reach the tumor and by “outnumbering” the immune suppressive mechanisms might overcome immune suppression and improve the anti-tumor response of ACT. Abbreviations: ACT—Adoptive cell therapy, CR—Chemokine receptor, MDSC—Myeloid derived suppressor cell, TAM—Tumor associated macrophage, TIL—Tumor Infiltrating lymphocyte (here representing anti-tumor T cells), Treg—Regulatory T cell.

The accumulating data supports the idea that chemokine receptor engineering of T cells as a strategy to improve homing and the potential response to treatment with ACT (illustrated in Figure 1). It is difficult to conclude whether one chemokine receptor is superior to any of the others in its property of improving tumor homing and, importantly, not home to anatomical sites other than the tumor. However, crucial for all is the local production of chemokine at the tumor site, which makes this approach very interesting in the personalization of cancer therapy. Of note, even though the term “personalization” is the current hype, the characterization of any one chemokine that could be used in the vast majority of patients maybe even across several indications, which would be of great value. To this end, chemokine receptor engineering has entered clinical testing. Based on data from the same group, a Phase I/II clinical trial of chemokine receptor engineered TILs, which combines lymphodepleting chemotherapy with ACT of TILs transduced with CXCR2 and NGFR (NCT01740557) and high dose IL-2, has been initiated at MD Anderson in Texas, USA. Data on the primary outcome is expected by January 2020. A main pitfall using TILs could be that the vast majority of T cells among TIL are not tumor-specific for which reason most T cells when attracted to the tumor would be expected to neither stay nor kill cancer cells. 

## 3. Disruption of Molecular Mediators of Homing

Several studies of T cell homing to tumors have used *in vivo* imaging using firefly luciferase transduction of T cells by enabling highly sensitive and specific tracking of T cells *in vivo* [74,75,76,77]. However, only very few studies have quantified the frequencies or total numbers of T cells in tumors [75,76,77]. Despite that most studies show that chemokine receptor engineered T cells can increase the frequencies of T cells infiltrating into the tumors after ACT in *in vivo* mouse models, the reported T cell homing is rather modest [3,74,75,76,78]. While IL-8 and other chemokines targeted through chemokine receptor engineering may induce the recruitment of T cells, T cell extravasation and migration into peripheral tissues rely on selectin and integrin binding in concert with chemokine signaling to mediate rolling and firm adhesion to the endothelial wall [1,38]. However, the vasculature of developing tumors may not necessarily support T cell adhesion, extravasation, and tumor infiltration [92]. 

Through a disproportionate expression of angiogenic cytokines and inhibitors, tumor vasculature is characterized by an abnormal structure and altered endothelia. This results in irregular blood flow, increased vascular permeability from leaky tumor vasculature, and insufficient lymphatic drainage [92,93,94]. These hypoxic neo-angiogenic vessels in the tumor are dysfunctional in the expression of homing molecules such as adhesion molecules (e.g., ICAM-1, VCAM-1, E- and P- selectins) and chemokines, and consequently less capable of supporting T cell flux and extravasation [95,96,97]. Tumor instigated chemokine/chemokine receptor mismatching, disrupted endothelial vessels, and adhesion of molecule expression are considered key factors in limiting TIL infiltration [1]. One strategy to tackle these obstacles may come from an unexpected source, which is exercise.

## 4. Facilitating Immune Infiltration through Exercise

Recent studies have linked the benefits of exercise on cancer risk and survival and an increased infiltration of immune cells into the tumor [98,99,100,101]. We recently studied the impact of exercise on tumor growth in mouse models taking advantage of voluntary wheel running, which was shown to delay tumor growth in transplantable models (B16 melanoma and Lewis lung) as well as tumor onset and tumor burden in a spontaneous model of melanoma and a decrease in the penetrance and delay in the onset of liver tumors using a chemically induced liver carcinoma model [101]. Detailed studies of B16 tumors showed a significant increase in the infiltration of effector immune cells including NK cells, T cells, and dendritic cells in tumors from exercising animals. Moreover, we could show that the release of epinephrine upon exercise led to the mobilization of immune cells—NK cells, T cells, and, to a lesser extent, B cells—into the circulation of the animals. Blocking this mobilization blocked the effect of exercise, which demonstrated that epinephrine-associated mobilization of immune cells is key to the effect. The same mechanism of mobilization of T cells and NK cells is well described in humans [102,103,104] and thought to represent the release of immune cells from secondary lymphoid organs (LNs and Spleen) and, through several physiological and molecular changes occurring during exercise, redistribute immune cells to the peripheral tissue.

During exercise, the parasympathetic nervous system directly controls the blood circulation, which ensures blood perfusion of vital organs and contracting muscle. In addition, adaptations to exercise include the angiogenesis and increased capillarization of the muscle [105,106,107]. Mechanisms of exercise might act in a similar manner on tumor vasculature and tumor perfusion. In mice with orthotopic mammary or prostate tumors, endurance training is associated with increased angiogenesis and vascularization of the tumors. This correlated with increased tumor blood perfusion and decreased tumor growth [36,108,109,110,111,112].

While increased blood perfusion of the tumor might confer a better oxidation and delivery of nutrients to the tumor, improved intra-tumoral vascularization and blood perfusion also confer an increased accessibility of circulating immune cells. In addition, improved perfusion limits intratumoral hypoxia [111,112], which is a factor associated with the suppression of anti-tumor immune responses in the tumor microenvironment [113,114,115,116,117]. Intratumoral hypoxia can affect the chemokine/chemokine receptor signalling axes [118,119,120] and have been shown to inhibit immune cell cytotoxicity [113,114,115,116,117]. In line with this, tumors from exercising mice display a diminished level of hypoxia and upregulation of chemokines [101]. The effect of hypoxia on anti-tumor CD8^+^ T cells are reviewed in detail by Vuillefroy de Silly 2016 [121]. Another physiologic change during exercise is the rise in core body temperature. In line with the above arguments, the temperature mediated dilation of peripheral vasculature (a mechanism of releasing excess heat from the body) could increase the access of circulating immune cells into tumors [122,123,124]. Induced hyperthermia increased intra-tumoral blood vessel diameters in mice with xenograft breast tumors [122]. In the same study, authors observed an increased infiltration of immune cells with hyperthermia and delayed tumor growth, suggesting that hyperthermia-induced blood vessel dilation increases the accessibility of immune cells. 

Hyperthermia hase been shown to modify the tumor vasculature by upregulating the adhesion of molecules E/P-selectins and ICAM-1 expression by inducing IL-6 trans-signalling on the endothelium [124,125]. This increased the CD8^+^ T cell adhesion and extravasation in a mouse model of B16 melanoma [124] and reduced tumor growth. In our exercise model, daily epinephrine injections mimicked the exercise-induced immune infiltration into tumors. However, it did not account for the full infiltrate observed with exercise [101]. One might speculate that our findings of an IL-6 dependent redistribution of immune cells to the tumor site [101] might be a result of hyperthermia facilitated accessibility and the induction of IL-6 trans-signalling with subsequent IL-6 dependent on the upregulation of adhesion molecules.

It has been speculated whether exercise can increase the functionality of tumors infiltrating immune cells. Data published in the late 1980s and 1990s showed that splenocytes from exercise-adapted mice mediated the increased killing of NK sensitive YAC-1 cells compared to splenocytes from control mice [126,127]. These studies use unsorted splenocytes and does not take into account the increase of a baseline level of peripheral NK cells in mice adapted to exercise [101,127,128]. Thus, the increase in killing could simply be accounted for by an increase in the number of NK cells in their killing assay. When using sorted NK cells, we [101] did not observe any difference in the killing of the NK sensitive YAC-1 cell line between exercise-adapted mice and control mice. Similarly, human NK cells isolated prior to and after exercise killed the NK sensitive human K562 cell line equally well [128]. However, it has been observed that exercise could increase the cytokine synthesis of IFNγ, IL-2, IL-12, and TNFα by murine lymphocytes restimmulated in vitro [129]. Thus, it could be contemplated whether exercise could support an anti-tumor response by modifying the cytokine secretion of lymphocytes.

The data discussed above suggests a potential role for exercise in cancer immune therapy. Despite this, no study, to our knowledge, have yet addressed the effect of exercise on the response to immune therapeutics (checkpoint inhibitors, ACT, vaccines, etc.).

**Figure 2:**
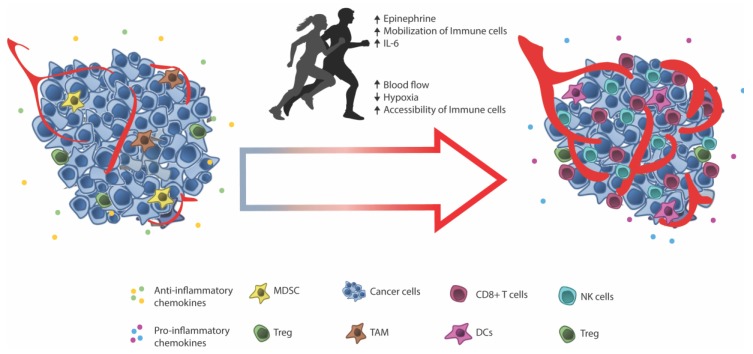
Exercise may improve the efficacy of immune therapy by several mechanisms attributed to the effect of physical activity. Exercise induced epinephrine mobilize immune cells (NK-cells, T-cells, and B cells) from the secondary lymphoid tissue, releasing them into circulation. The concurrent increase in blood flow, through vasodilation and normalization of tumor vasculature, increase the accessibility and entrance of immune cells into the tumor. Among the benefits of increased perfusion is a decrease in hypoxic areas thought to modulate the effector function of infiltrating immune cells and expression of pro-inflammatory chemokines, which further aids the infiltration of more anti-tumor immune cells. Exercise could, therefore, be prescribed prior to ACT to increase the TIL numbers prior to biopsy and *in vitro* expansion as well as following ACT to secure the homing and on-site function of transferred T cells. Abbreviations: DC—Dendritic cell, IL—Interleukin, MDSC—Myeloid derived suppressor cell, NK—Natural killer, TAM—Tumor associated macrophage, and Treg—Regulatory T cell.

Prescribing exercise to cancer patients prior and during treatment with immune therapy represents an interesting non-invasive strategy that might facilitate a better response to therapy (See Figure 2). To this end, exercise prior to TIL therapy could potentially improve the quality and quantity of TILs harvested from tumors. In addition, one might speculate whether a continued exercise regimen after TIL transfer may improve tumor localization and functioning on site of the TILs. While exercise is currently prescribed and evaluated in patients with metastatic cancer (NCT02159157, and more www.clinicaltrials.gov), the primary endpoint is not to assess immune infiltration and response to immune therapy but rather the physical functioning of patients [130]. Thus, prospective clinical trials are needed to fully elucidate the exercise-induced immune infiltration in cancer patients. Considering the non-invasive nature of exercise, it should be evaluated exactly how much (or how little) exercise is needed for TIL infiltration and improved TIL harvest. In addition, assessing exercise in combination with checkpoint inhibitors is of great interest since it is becoming increasingly clear, that patients with low or no T cell infiltrate prior to therapy are less likely to respond to therapy [131].

## 5. Perspectives and Concluding Remarks

In light of the current knowledge, increasing the homing of effector T cells is crucial for ACT. Current strategies are addressing the issue of homing by genetically engineering T cells with chemokine receptors, which have shown promising preclinical results. However, fixing the T cells with GPS coordinates of the tumor does not fix the other road blocks. In this paper, we hypothesized a role for exercise in normalizing the poor tumor vascularization and lack of appropriate adhesion molecules and creating highways to the tumor.

Exercise has no adverse effects, but it leads to healthier living, decreases systemic inflammation as well as blood pressure and pre-diabetic states and increases physical functioning. In addition, it can be applied prior to ACT to increase the number of TILs in biopsies or increase the number/quality of peripheral blood cells for harvest in CAR/TCR therapy as well as after ACT where it can increase the accessibility and infiltration into remaining tumor lesions. Looking outside of the scope of improving ACT, one might speculate that getting more immune cells into tumors by exercise could assist other strategies of immune therapy. Checkpoint inhibitors relies on pre-existing tumor immune infiltration since patients with so called “cold” tumors fail to respond to checkpoint inhibitors [9,132,133]. Exercise might be a means to tip the balance by improving the response rate and depth of responses.

## Figures and Tables

**Table 1:** Excerpt of chemokine/chemokine receptors involved in the homeostatic and inflammatory adaptive T cell immune response and function in tumor immunology. More than 50 chemokines and 20 chemokine receptors have been described. Therefore, this table is limited to the migration axes described in the introduction section and the 11 among which this thesis revolves around (highlighted in bold font). Abbreviations: DC, dendritic cell, ILC, Innate lymphoid cells, LN, lymph node, MDSC, myeloid derived suppressor cells, NK, Natural Killer cells, NKT, Natural killer T cells, TAM, tumor associated macrophages, TAN, tumor associated neutrophils, Th, Thelper, TLS, Tertiary lymphoid structures, Treg, regulatory T cell. The role of chemokines in pro-tumor and anti-tumor responses have been reviewed in detail by Viola et al 2012 [13]. This table is a modified merger from Viola et al. 2012 [13] and Griffith et al. 2014 [38].

Chemokine	Alternative Name	Receptor(s)	Function an Adaptive T Cell Response	Function in Tumor Immunology
CCL2	MCP-1	CCR2, CCR3	Inflammatory monocyte trafficking	Recruitment of TAMs, MDSC, and neutrophils, Tumor infiltration of CD8^+^ T cells
CCL3	MIP-1α	CCR5	T cell-DC interactions	Recruitment and maturation of DCs, increased CD8^+^ T cell activation and tumor infiltration
CCL4	MIP-1β			
CCL5	RANTES			
CCL17	TARC	CCR4	Treg migration, Th2 response and migration	Treg recruitment and tumor infiltration
CCL19	MIP-3β	CCR7	T and DC homing to LNs	Formation of tumor associated TLS, recruitment and activation of Treg and MDSC, tolerogenic TLS and promotion of metastasis
CCL21	SLC			
CCL22	MDC	CCR4	Treg migration, Th2 response and migration	Treg recruitment and tumor infiltration
CCL25	TECK	CCR9	T cell precursor homing to thymus	Inhibit effector T cell function, chemotherapy resistance, and metastasis [56]
CXCL1	Gro-α	CXCR1, CXCR2	Neutrophil trafficking	Survival, proliferation, and metastasis (cancer cells), Neoangiogenesis, recruitment of MDSC and TANs
CXCL8	IL-8	CXCR1, CXCR2,		
CXCL9	MIG	CXCR3	Th1 response, CD8, Th1 and NK trafficking to site of inflammation	Recruitment and infiltration of CD8^+^ T, NK, and NKT cells, inhibit tumor cell proliferation, vascular transmigration checkpoint [14]
CXCL10	IP-10			
CXCL11	I-TAC			
CXCL12	SDF-1	CXCR4	Bone marrow homing, LN homing	MDSC recruitment and promotion of metastasis
CXCL16	LEC, etc.	CXCR6	NKT and ILC migration and survival	Recruitment of activated T cells, NK cells, and monocytes
